# Genomic selection in sugar beet breeding populations

**DOI:** 10.1186/1471-2156-14-85

**Published:** 2013-09-18

**Authors:** Tobias Würschum, Jochen C Reif, Thomas Kraft, Geert Janssen, Yusheng Zhao

**Affiliations:** 1 State Plant Breeding Institute, University of Hohenheim, 70593 Stuttgart, Germany; 2 Syngenta Seeds AB, Box 302, 261-23 Landskrona, Sweden; 3 Present address: Leibniz Institute of Plant Genetics and Crop Plant Research (IPK), 06466 Gatersleben, Germany; 4 Present address: Bayer Vegetable Seeds, 40019 Sant' Agata Bolognese, Italy

## Abstract

**Background:**

Genomic selection exploits dense genome-wide marker data to predict breeding values. In this study we used a large sugar beet population of 924 lines representing different germplasm types present in breeding populations: unselected segregating families and diverse lines from more advanced stages of selection. All lines have been intensively phenotyped in multi-location field trials for six agronomically important traits and genotyped with 677 SNP markers.

**Results:**

We used ridge regression best linear unbiased prediction in combination with fivefold cross-validation and obtained high prediction accuracies for all except one trait. In addition, we investigated whether a calibration developed based on a training population composed of diverse lines is suited to predict the phenotypic performance within families. Our results show that the prediction accuracy is lower than that obtained within the diverse set of lines, but comparable to that obtained by cross-validation within the respective families.

**Conclusions:**

The results presented in this study suggest that a training population derived from intensively phenotyped and genotyped diverse lines from a breeding program does hold potential to build up robust calibration models for genomic selection. Taken together, our results indicate that genomic selection is a valuable tool and can thus complement the genomics toolbox in sugar beet breeding.

## Background

Genomic selection has been suggested as a novel approach to increase selection gain in crop and livestock breeding programs [1-3]. Whereas QTL mapping strategies are based on the assumption that individual chromosomal regions can be identified that contribute to the trait and whose effects are estimated, genomic selection uses genome-wide marker data to estimate genomic breeding values of individuals. For plant breeding, genomic selection has been evaluated using empirical data from different crops, including maize e.g., [4-11], barley e.g., [12-14], wheat e.g., [5,15-17], as well as sugar beet [18].

Ridge regression best linear unbiased prediction (RR-BLUP) [1,19] has been shown to provide high prediction accuracies across a range of crops and traits [14]. RR-BLUP assumes that each marker contributes to the trait and has the same variance which is in accordance with the infinitisemal model of quantitative genetics and explains why RR-BLUP provides good results for complex traits [20]. Genomic selection is based on linkage disequilibrium between markers and QTL affecting the trait. In addition, Habier et al. [21] showed that the accuracy of genomic selection depends on the exploitation of genetic relationships between individuals. RR-BLUP was most efficient in exploiting these genetic relationships since all available markers are used in the model. Plants within breeding programs will always show a certain degree of relatedness and in addition, most important agronomic traits are complex traits. This suggests that RR-BLUP should be well suited for genomic selection in applied plant breeding. Another major advantage of RR-BLUP is that it is computationally less demanding than other approaches.

In genomic selection marker effects are first estimated based on a set of individuals which have been phenotyped and genotyped. This is often referred to as the training population. In a second step, the breeding values of individuals that have been genotyped but not phenotyped are predicted. It has been shown that the prediction accuracy decreases when the genetic relatedness between the individuals in the training population and those in the prediction set decreases [21] and that high accuracies require that genotypes from the populations in which prediction will be done are represented in the training population [22]. In applied plant breeding programs different germplasm types are available: large biparental families from early generations which have not been selected yet and which are tested less intensively, and diverse lines from late generations that remained after several rounds of selection [23]. The latter are tested most intensively in field trials and are often also genotyped to characterize them at the molecular level. A key question for an efficient and cost-effective implementation of genomic selection in breeding programs is therefore whether a calibration model developed based on a training population consisting of a diverse set of lines can be used for prediction of the phenotypic performance within segregating families.

In this study we employed a large sugar beet population consisting of a panel of diverse lines and four segregating families to evaluate the potential of genomic selection for different yield- as well as quality-related traits in sugar beet and to investigate the prediction accuracy of genomic selection within families using a training population composed of a diverse set of lines.

## Results

**Figure 1:**
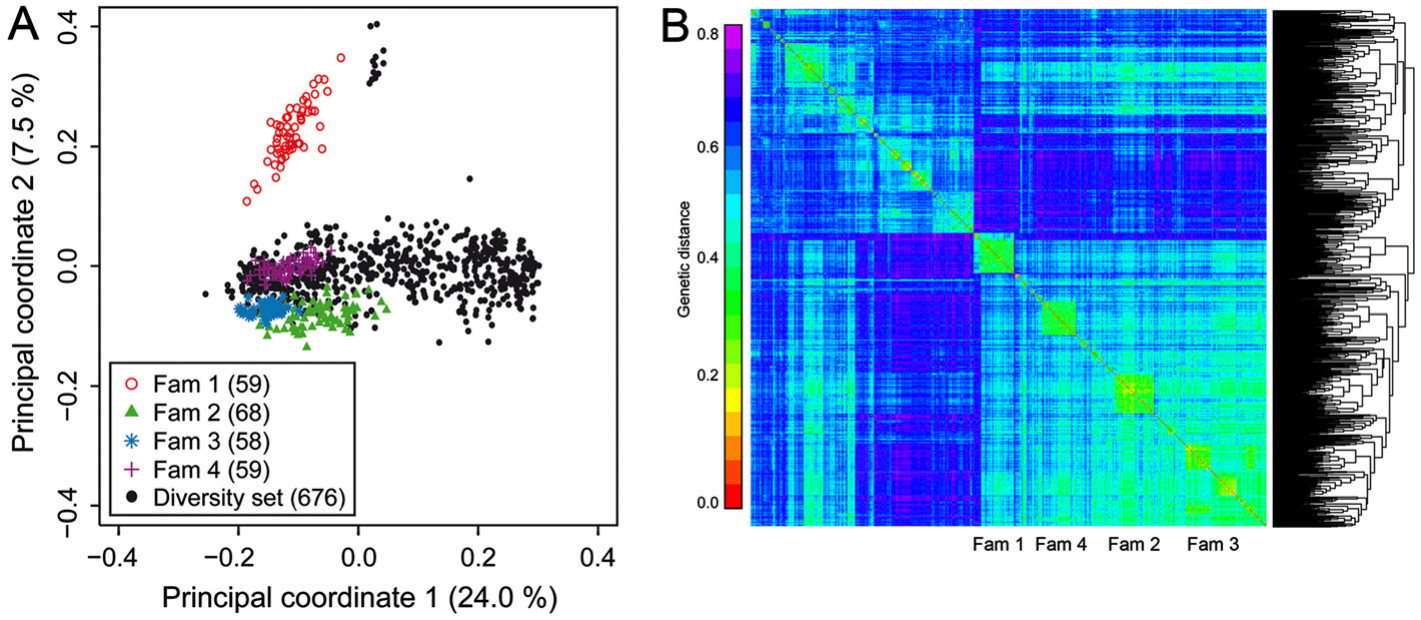
**Genetic kinship. (A)** Principal coordinate analysis of the 924 genotypes based on modified Rogers' distance estimates. The diversity set and the four biparental families are shown. The numbers in brackets refer to the proportion of variance explained by the principal coordinate. **(B)** Genetic kinship among the 924 genotypes. Average linkage clustering was used to order the distance matrix. The four families are indicated below the plot.

**Figure 2:**
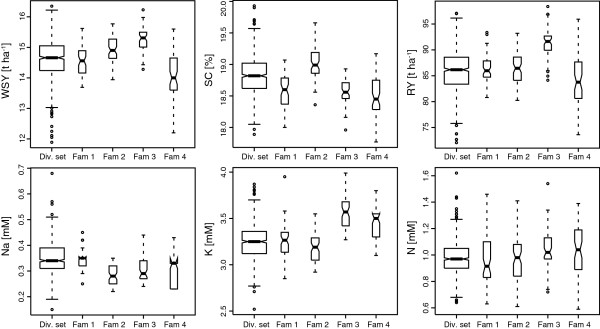
**Box-Whisker-Plots for the six traits shown for the diversity set and for each of the four families.** The width of the plots is proportional to the number of individuals in each group and the notches provide evidence whether or not two medians differ. White sugar yield (WSY), sugar content (SC), root yield (RY), sodium content (Na), potassium content (K), and α-amino nitrogen content (N).

The population under study is composed of a total of 924 lines which can be divided into two subpopulations: 248 lines are derived from four biparental families that are connected by one common parent and 676 lines form a diversity set with different degrees of relatedness (Figure 1). All six traits showed significant genotypic variance estimates (*P* < 0.01) and medium to high heritabilities in the entire population (0.38 to 0.71) and in the diversity set (0.51 to 0.70) while across the four families the heritabilities ranged from 0.24 to 0.76 (Additional file 1: Table S1). In single families the heritabilities ranged between 0.02 to 0.60. The Box-Whisker-Plots indicate significant differences among the four families for all traits (Figure 2). Consequently, the data set presents a good basis to evaluate the prospects of genomic selection in applied sugar beet breeding.

**Figure 3:**
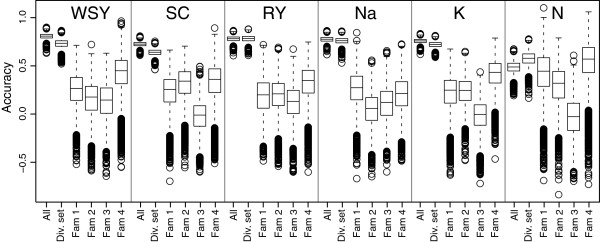
**Box-Whisker-Plots for the accuracy of genomic predictions assessed by fivefold cross-validation.** Results are shown for the entire population, the diversity set, and the four families, for white sugar yield (WSY), sugar content (SC), root yield (RY), sodium content (Na), potassium content (K), and α-amino nitrogen content (N).

We used fivefold cross-validation to assess the accuracy of genomic predictions for the six traits in the entire population and in the diversity set (Figure 3). We found that the cross-validated prediction accuracy was high for all traits except for α-amino nitrogen content in the entire population which showed only a moderate prediction accuracy. The highest prediction accuracy was observed for white sugar yield. The prediction accuracies in the four families were generally lower than those obtained in the diversity set. It must be noted here, that for comparison the variance component and heritability estimates of the entire population were used also for the families. To exclude the possibility that the obtained prediction accuracies were affected by the unbalanced phenotyping, we repeated the fivefold cross-validation with a subset of lines that has been evaluated in at least three locations. The prediction accuracies were comparable to those of the full data set (Additional file 1: Figure S1). Consistent with previous studies [8,15], we observed that an increase in population size or marker density resulted in an increased prediction accuracy with less variation across cross-validation runs (Additional file 1: Figure S2).

**Figure 4:**
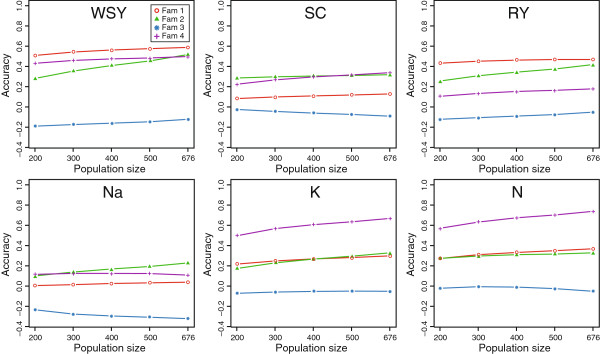
**Accuracy of genomic predictions for effect estimation in the diversity set and prediction in individual families.** Results are shown for different numbers of plants sampled from the diversity set for effect estimation. White sugar yield (WSY), sugar content (SC), root yield (RY), sodium content (Na), potassium content (K), and α-amino nitrogen content (N).

We next evaluated the accuracy obtained by estimating marker effects in the diversity set and predicting the breeding values in each of the four families (Figure 4). We found that the prediction accuracy varied among the families and was even negative for Family 3. With the exception of α-amino nitrogen content in Family 4, the prediction accuracies were substantially lower than those obtained within the diversity set (Figure 3). We then varied the size of the training population by randomly selecting different numbers of individuals from the diversity set for prediction in the families. For most traits and families the prediction accuracy decreased slightly with decreasing individuals in the training set (Figure 4).

## Discussion

### Genomic selection in sugar beet breeding

Sugar beet is well suited for genomic research as many SNP markers are already available and the assembly of a draft genome sequence is approaching completion. In the entire population which represents typical breeding germplasm, we observed high cross-validated prediction accuracies between 0.72 and 0.80 for all traits except for α-amino nitrogen content which only showed an accuracy of 0.48 (Figure 3). This may be attributed to the low heritability of this trait. Hofheinz et al. [18] recently reported the application of genomic selection in sugar beet. Based on a population consisting of 310 inbred lines derived from 34 crosses they obtained prediction accuracies for sugar content and loss to molasses of 0.82 and 0.86, respectively. We investigated two additional yield-related and three quality-related traits and overall our results on the accuracy that can be achieved for complex traits in sugar beet are in agreement with those reported in the previous study of Hofheinz et al. [18]. Our results are also in agreement with those for complex traits in maize and wheat for which prediction accuracies of comparable magnitude have been reported [6,8,14-16,24].

A recent association mapping approach in this population identified QTL for all six traits but only few QTL explained a proportion of the genotypic variance sufficiently high to warrant a marker-assisted selection approach [25]. This suggests that the traits studied here must all be regarded as complex traits that are mainly controlled by many small effect QTL. These will escape detection in QTL mapping approaches but their effects can be captured in genomic selection. It must be noted here, that the prediction accuracy in applied plant breeding will decrease across cycles as allele frequencies change, QTL become fixed, and novel QTL alleles are introduced. Thus, genomic selection represents a promising and powerful genomics tool for sugar beet breeding but will require a constant recalibration of the prediction models.

### Training populations in applied plant breeding

In plant breeding programs, late generation lines representing the future varieties are tested most intensively in multi-location field trials and are often also characterized with molecular markers [23]. Consequently, these lines represent the most obvious training population that could be used in applied plant breeding as both, high-quality phenotypic data and genotypic data are available for these lines. Our results revealed high prediction accuracies within such a diversity set (Figure 3). However, as these lines are subjected to intensive field testing anyhow, the potential of genomic selection with these lines lies in the prediction of traits that are difficult to score or that do not occur regularly and are therefore difficult to phenotype conventionally (e.g., certain quantitative resistances or abiotic stress). Genomic selection could then be applied to late generation lines for such traits to reduce resource-intensive phenotyping.

### Selection within segregating families

In applied plant breeding new crosses are initiated every year and an important step is the selection of lines within these segregating families. The question that arises is whether the above mentioned training population consisting of a diversity set can be used for the prediction within families. This might represent an interesting complement to phenotypic selection for traits with high genotype times environment interactions. Applying this approach, we observed that the prediction accuracy was lower than that observed in the diversity set and varied among families (Figure 4). There was, however, no consistent trend with regard to their ranking and the prediction accuracy was not affected by the genetic relatedness of the single families to the diversity set. Family 2 was on average most closely related to the diversity set, but did not show a higher accuracy of genomic predictions as compared to the other families (Additional file 1: Table S2 and Figure S3). It must be noted, however, that the average genetic distances to the diversity set were not much different between families. A possible explanation for the observed differences in the ranking between families is that the accuracy of genomic predictions in the families will be high if similar QTL are segregating in a family and in the diversity set, and if QTL alleles have comparable effects. As this will vary between traits, the prediction accuracy will also vary and consequently the accuracy that can be expected in any of the families for a given trait cannot be predicted beforehand.

In general, the prediction accuracies obtained in the families for effect estimation in the diversity set were lower than those in the diversity set. Potential reasons for this decrease in accuracy include but are not limited to: (i) Insufficient size of the training population. We did observe a dependency on the size of the training population as the accuracy decreased with fewer plants sampled from the diversity set. However, this effect was rather small and the potential to further increase the accuracy within families by increasing the size of the training population composed of diverse lines was limited in this study. (ii) The genetic architecture. The number and the effect sizes of the QTL may differ between the diversity set and families. In addition, epistasis (QTL-times-genetic background interactions) which has been shown to affect these traits in the population under study [25] may contribute to the traits to a different extent. (iii) Different extent of LD between the markers and QTL. Especially for markers that are more distant from the QTL the LD may vary [25]. In addition, the markers closest to the QTL may not be segregating in the families making prediction less accurate (Additional file 1: Figure S4). High marker densities that are available nowadays at competitive costs for most crops can restrict the effect of LD since a high genome coverage ensures a consistently high LD between markers and QTL in each population. (iv) Multiple alleles. SNPs markers are usually biallelic and can thus only distinguish two alleles. If multiple alleles at a QTL are present and the QTL is linked to a SNP, then one SNP allele may be linked to two or more different QTL alleles. Therefore, the presence of identical SNP alleles in two plants does not necessarily imply identical QTL alleles. (v) Genetic distance. RR-BLUP achieves its accuracy mainly by efficiently exploiting genetic relationships between individuals and the accuracy has been shown to decrease with increasing genetic distance between the training and prediction set [21]. However, since the parents of the families were derived from the same breeding pool and are related to the lines from the diversity set, the lines within families are also not entirely unrelated to the diversity set (Figure 1). The reduction in accuracy observed here may thus be attributed to combinations of the above mentioned reasons.

The negative accuracies observed for Family 3 are intriguing and similar results have recently been reported for prediction among less related biparental families in maize [26]. Notably, the prediction accuracy for Family 3 showed the same trend as observed for the other families, as the accuracy often became increasingly stronger (albeit negative) with increasing size of the training population. This suggests that the training population provided a negative prediction signal which points to opposite linkage phases between markers and important QTL in the diversity set and in Family 3. Consistently, findings from a simulation study showed that the combination of families into a training population requires persistence of marker-QTL LD across these families [22]. It must be noted, that this information may not be captured by the genome-wide estimates as they do not necessarily reflect the similarities or differences at the essential QTL regions. In addition, as indicated by the principal coordinate plot, Family 3 possessed the lowest within-family genetic variation of all families and we observed a strong correlation between this within-family genetic variation and the obtained prediction accuracies (Additional file 1: Table S2). A lower genetic variation will result in a lower variation of predicted phenotypes. As this within-family genetic variation was not mirrored in the phenotypic variation of the families (Additional file 1: Table S1), this may be another cause for the low or even negative prediction accuracies. Furthermore, we observed a higher number of monogenic markers in Family 3 which in contrast to the other families may leave important QTL regions without segregating markers (Additional file 1: Figure S4). The example of Family 3 illustrates that the prediction in families for effect estimation in a diversity set may not always work and requires a careful choice of the lines used as training population. Only if the training population is representative for the family in which prediction is to be done, genomic selection can be advantageous with high prediction accuracies.

**Figure 5:**
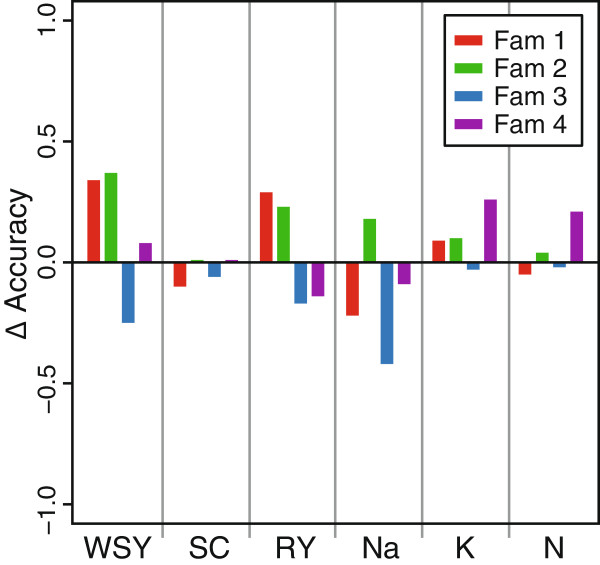
**Prediction accuracy in the four families.** Difference in prediction accuracy in each family shown for the six traits for effect estimation in the diversity set and prediction in the families as compared to fivefold cross-validation in the respective family. White sugar yield (WSY), sugar content (SC), root yield (RY), sodium content (Na), potassium content (K), and α-amino nitrogen content (N).

With some exceptions the prediction accuracies in the families were not high, but nevertheless at a level where genomic selection could be attractive for preselection in early stages to screen many genotypes with less accuracy. It must be noted, however, that in order to enable a comparison the heritability estimates from the entire population were used. Standardizing with the lower heritability estimates of the families to obtain *r*_*GS*_ would increase the obtained prediction accuracies. Likewise, the obtained cross-validated accuracies within the families were also rather low (Figure 3). The prediction accuracies in the families when effect estimation was performed in the diversity set were often as high or even higher than the ones obtained by cross-validation in the respective family (Figure 5).

## Conclusions

We have shown that genomic selection is a promising genomic approach for sugar beet breeding. In addition, we present an approach of using a set of diverse lines as training population for prediction within segregating families. The obtained prediction accuracies were promising and show that this approach does hold some potential for application in applied plant breeding and warrants further research.

## Methods

### Plant materials, field experiments, and molecular markers

This study was based on 924 diploid elite sugar beet (*Beta vulgaris* L.) inbred lines which have been described before by Würschum et al. [25]. The lines are all pollinators with approximately equal proportions of S1, S2 and S3 inbred lines. The population consists of 676 diverse lines (the diversity set) and four biparental families which combined constitute 248 individuals (Figure 1). Testcross progenies were produced by crossing the genotypes to the same single-cross hybrid as tester. All material used in this study was provided by the breeding company Syngenta Seeds AB (Sweden).

The 924 genotypes were evaluated in routine plant breeding trials in 2008 with two replicates at 1–7 locations in Europe (Additional file 1: Table S3). The evaluated traits were white sugar yield (WSY, t ha^-1^), sugar content (SC, %), root yield (RY, t ha^-1^), potassium content (K, mM), sodium content (Na, mM), and α-amino nitrogen content (N, mM).

The 924 genotypes were fingerprinted following standard protocols with 677 single nucleotide polymorphism (SNP) markers that are polymorphic in the population under study. These markers were randomly distributed across the sugar beet genome with an average marker distance of 1 cM and a maximum gap between adjacent markers of 23 cM. Map positions of all markers were based on the linkage map of Syngenta Seeds AB with a total map length of 698 cM. Following the suggestion of Crossa et al. [5] missing marker genotypes were imputed with probabilities corresponding to the respective allele frequencies.

### Phenotypic data analyses

The analyses were based on adjusted entry means calculated for each location. The following linear mixed model was used to estimate variance components of the testcrosses: *y*_*ij*_ ~ *μ* + *l*_*j*_ + *g*_*i*_ + *e*_*ij*_, where *y*_*ij*_ is the adjusted entry mean of the *i*th sugar beet line at the *j*th location, *μ* the intercept term, *l*_*j*_ the effect of the *j*th location, *g*_*i*_ the genetic effect of the *i*th sugar beet line, and *e*_*ij*_ the error term including the genotype times location interaction effect. Locations and genotype were modeled as random effects. Variance components were determined by the restricted maximum likelihood (REML) method. Significance for variance component estimates was tested by model comparison with likelihood ratio tests where the halfed P values were used as an approximation [27]. As for testcross progenies with one common tester the variance components due to general and specific combining ability effects cannot be estimated independently [6] we used the heritability on an entry-mean basis being aware that this might result in an underestimation of the prediction accuracy. Furthermore, genotypes were regarded as fixed effects and best linear unbiased estimates (BLUEs) were determined for all genotypes and traits.

Associations among the 924 genotypes were analyzed by applying principal coordinate analysis (PCoA) [28] based on the modified Rogers’ distances [29]. PCoA computations were performed with the software package Plabsoft [30].

### Genomic selection

Genomic selection was done by ridge-regression BLUP (RR-BLUP) [4,19,31]. For RR-BLUP, the following model was used to obtain estimates of the marker effects $y=\mu +\sum_{j=1}^{N_{m}}X_{j}a_{j}+e$, where *y* is a *N* x 1 vector of BLUEs estimated across locations; *N*_*m*_ refers to the number of fitted markers; *a*_*j*_ is the effect of the *j*th marker; *X*_*j*_ is a *N* × 1 vector denoting the genotype (coded as 0-1-2) of the individuals for marker *j*. Following the suggestion of Meuwissen et al. [1], we assumed that the variance of *a*_*j*_ is $\sigma_{G}^{2}$/*N*_*m*_. For a more detailed discussion see Gianola et al. [32]. We used the error variance of the BLUEs across locations, i.e., $\sigma_{e}^{2}$, divided by the number of locations (L) to derive the penalty parameter *λ*. Consequently, *λ* was defined as ($\sigma_{e}^{2}$/L) / ($\sigma_{G}^{2}$/*N*_*m*_). The estimates of *a*_*j*_ were obtained from mixed-model equations [33]. Given the estimates of *a*_*j*_ and the marker genotypes, genetic values were predicted as, $PV_{i}=\sum_{j=1}^{N_{m}}X_{\mathrm{ij}}{â}_{j}$ where *X*_*ij*_ is the marker genotype of individual *i* for marker *j*, and *â*_*j*_ is the estimated effect of marker *j*.

### Cross-validation

For the fivefold cross-validation, the respective data set was randomly divided into five subsets. Four of the subsets (80%) were used for the estimation of marker effects and the remaining subset (20%) was used as validation set. In the validation set phenotypic values were estimated based on the estimated marker effects as described above. The correlation between observed and predicted phenotypes (*r*_MP_) was calculated. The accuracy of genomic selection was expressed as *r*_GS_ = *r*_MP_ / *h*[34,35], where *h* refers to the square root of heritability. The sampling of training and validation sets was always repeated 10,000 times.

## Authors’ contributions

TK, GJ collected data, TW, YZ performed analyses, TW, JCR, YZ wrote the manuscript. All authors read and approved the final manuscript.

## Supplementary Material

Additional file 1 **Supplementary Information.** This file contains the Supplementary Tables and Figures. Click here for file
