# Supplementary material for: Genomic selection in sugar beet breeding populations
Source: BMC Genet. 2013 Sep 18;14:85. doi: 10.1186/1471-2156-14-85 (PMC3848454; doi:10.1186/1471-2156-14-85)
Supplement: Additional file 1 — Supplementary Information. This file contains the Supplementary Tables and Figures. [file 1471-2156-14-85-S1.pdf]

Genomic selection in sugar beet breeding populations

Tobias Würschum[1§], Jochen C Reif[1*], Thomas Kraft[2], Geert Janssen[2#], Yusheng Zhao[1*]

[1] State Plant Breeding Institute, University of Hohenheim, 70593 Stuttgart, Germany

[2] Syngenta Seeds AB, Box 302, 261-23 Landskrona, Sweden

* Present address: Leibniz Institute of Plant Genetics and Crop Plant Research (IPK), 06466 Gatersleben, Germany

# Present address: Bayer Vegetable Seeds, 40019 Sant'Agata Bolognese, Italy

§ Corresponding author

Additional files

Table S1 Variance component estimates and heritabilities. Genotypic variances (σ^2_G), error variance (σ^2_e; confounded with genotype times location interaction variance) and heritabilities (h^2) in the entire population, the diversity set and in the four families, for white sugar yield (WSY), sugar content (SC), root yield (RY), sodium content (Na), potassium content (K), and α-amino nitrogen content (N). Fam 4 has only been evaluated at one location for four of the traits, therefore no h^2 estimable.

	All genotypes	Diversity set	Families	Fam 1	Fam 2	Fam 3	Fam 4
WSY							
σ^2_G	0.18	0.21	0.10	0.03	0.03	0.003	-
σ^2_e	0.42	0.35	0.55	0.47	0.48	0.54	-
h^2	0.51	0.56	0.37	0.15	0.16	0.02	-
SC							
σ^2_G	0.06	0.06	0.06	0.02	0.02	0.02	0.02
σ^2_e	0.09	0.07	0.13	0.13	0.15	0.10	0.18
h^2	0.60	0.66	0.57	0.35	0.23	0.39	0.33
RY							
σ^2_G	8.18	8.42	7.93	0.91	3.04	4.13	5.70
σ^2_e	16.60	11.48	24.30	17.27	15.88	18.87	45.78
h^2	0.55	0.61	0.51	0.14	0.37	0.47	0.27
Na							
σ^2_G	0.002	0.002	0.001	0.0004	0.0003	0.0011	-
σ^2_e	0.004	0.004	0.003	0.0039	0.0028	0.0040	-
h^2	0.53	0.51	0.52	0.23	0.27	0.52	-
K							
σ^2_G	0.03	0.02	0.04	0.02	0.01	0.02	-
σ^2_e	0.03	0.02	0.04	0.04	0.03	0.04	-
h^2	0.71	0.70	0.76	0.60	0.56	0.64	-
N							
σ^2_G	0.008	0.009	0.007	0.008	0.007	0.005	-
σ^2_e	0.030	0.010	0.067	0.082	0.065	0.063	-
h^2	0.38	0.65	0.24	0.22	0.26	0.26	-

Table S2 Genetic distance between family lines and the diversity set, genetic distance variation within families and prediction accuracy for effect estimation in the diversity set. The correlation between the genetic distances and the obtained prediction accuracies and those between the genetic distance variation within each family and the obtained prediction accuracies are shown. White sugar yield (WSY), sugar content (SC), root yield (RY), sodium content (Na), potassium content (K), and α-amino nitrogen content (N).

	Genetic distance $GD_{Div\text{-}Fam}$	Genetic distance variation GD_{Var}	WSY r_{GS}	SC r_{GS}	RY r_{GS}	Na r_{GS}	K r_{GS}	N r_{GS}
Family 1	0.32	0.001095881	0.59	0.13	0.47	0.04	0.30	0.37
Family 2	0.27	0.000898929	0.52	0.32	0.42	0.23	0.33	0.33
Family 3	0.29	0.000588792	-0.12	-0.09	-0.05	-0.32	-0.05	-0.05
Family 4	0.31	0.000982229	0.50	0.34	0.18	0.11	0.67	0.74
Correlation $GD_{Div\text{-}Fam}$ - r_{GS} (*P* value)			0.50 (0.50)	-0.07 (0.93)	0.10 (0.90)	-0.10 (0.90)	0.33 (0.67)	0.41 (0.59)
Correlation GD_{Var} - r_{GS} (*P* value)			0.98 (0.01)	0.67 (0.33)	0.84 (0.16)	0.76 (0.24)	0.73 (0.27)	0.76 (0.24)

Table S3 Overview of plant breeding trials. Number of genotypes which have been evaluated at the given numbers of locations.

	Number of test locations					
	1	2	3	4	5	7
Entire population	292	119	393	97	19	4
Diversity set	292	119	204	38	19	4
Fam 1			60			
Fam 2			69			
Fam 3				59		
Fam 4			60			

The genotypes were tested at the following locations:
Artenay, FR; Burley, ES; Bury St. Edmunds, GB; Bystrice, CZ; Christianssaede, DK; Gross Mehring, DE; St Hilaire, FR; St Just, FR; Köpingebro, SE; Mourlancourt, FR; Moosham, DE; Marqueglise, FR; Sud de Paris, FR; Presles, FR; Santerre, FR; Stechworth Estates, GB; Vilhelmsdal, Falster, DK

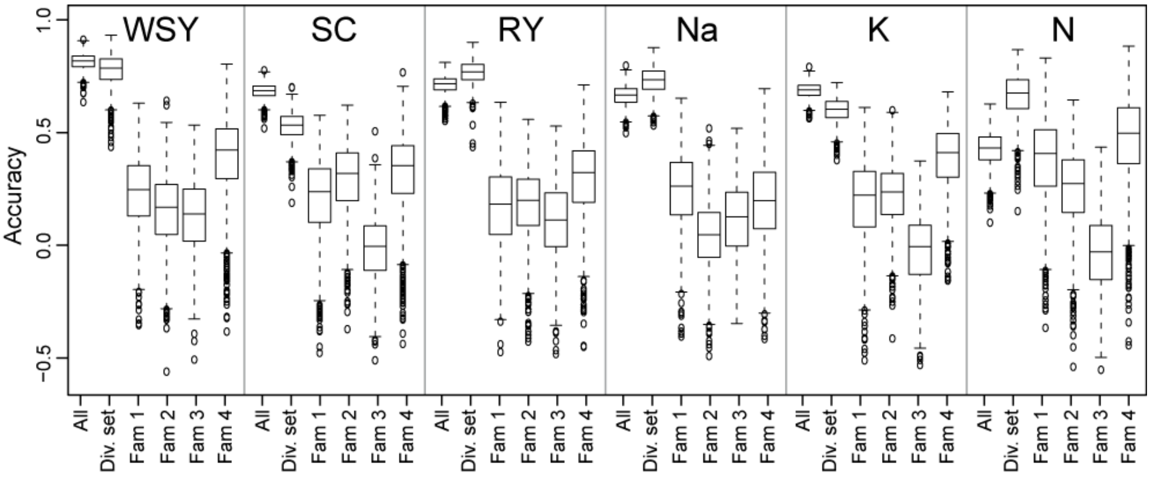

Figure S1 Box-Whisker-Plots for the accuracy of genomic predictions assessed by fivefold cross-validation. Results are based on a subset of lines (N = 513) that have been tested in at least three locations to exclude an effect of the phenotyping intensity on the obtained prediction accuracies. White sugar yield (WSY), sugar content (SC), root yield (RY), sodium content (Na), potassium content (K), and α-amino nitrogen content (N).

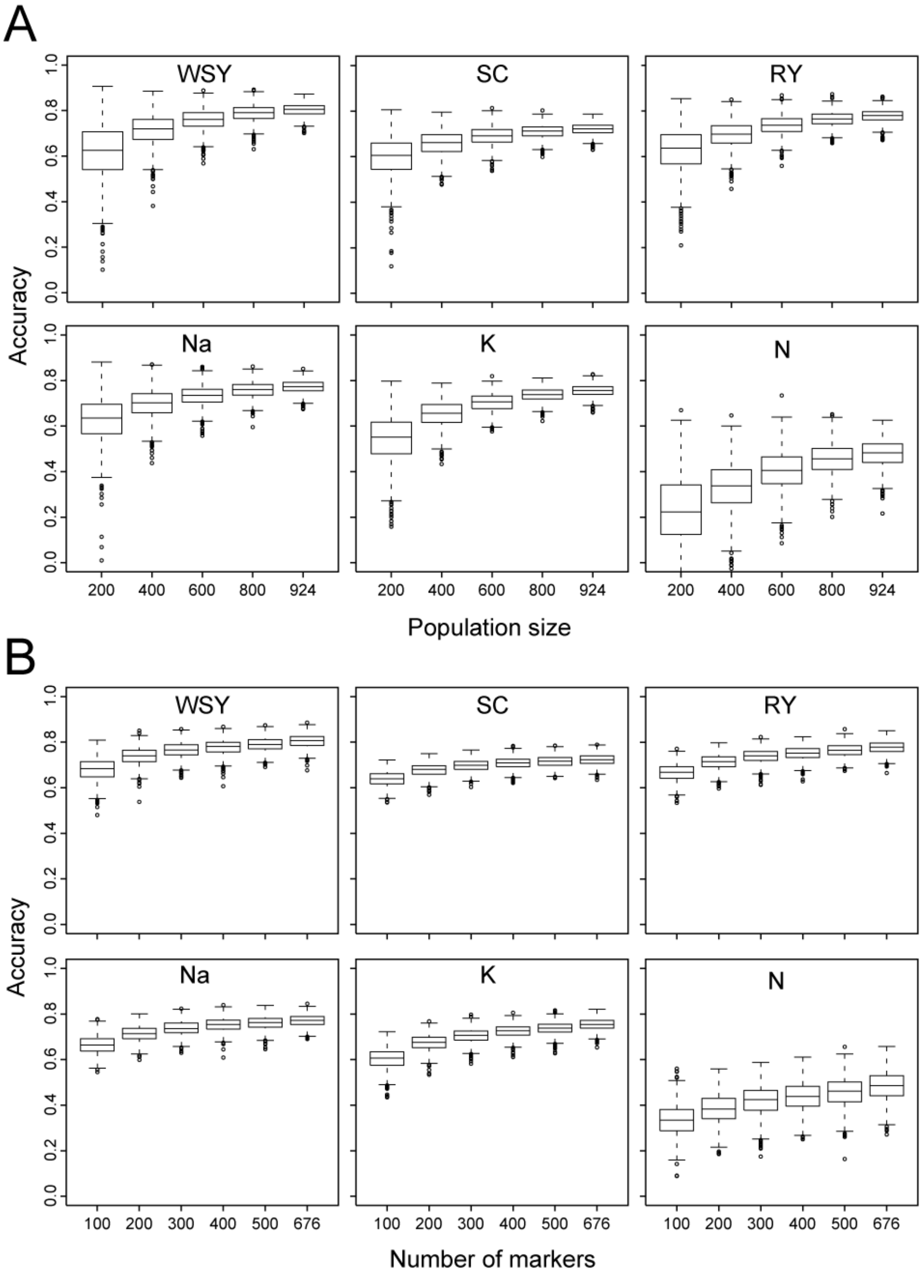

Figure S2 Effect of population size and marker density on the prediction accuracy. Box-Whisker-Plots showing the median and the variation in prediction accuracy assessed by fivefold cross-validation for (A) varying population sizes and (B) varying marker densities. White sugar yield (WSY), sugar content (SC), root yield (RY), sodium content (Na), potassium content (K), and α-amino nitrogen content (N).

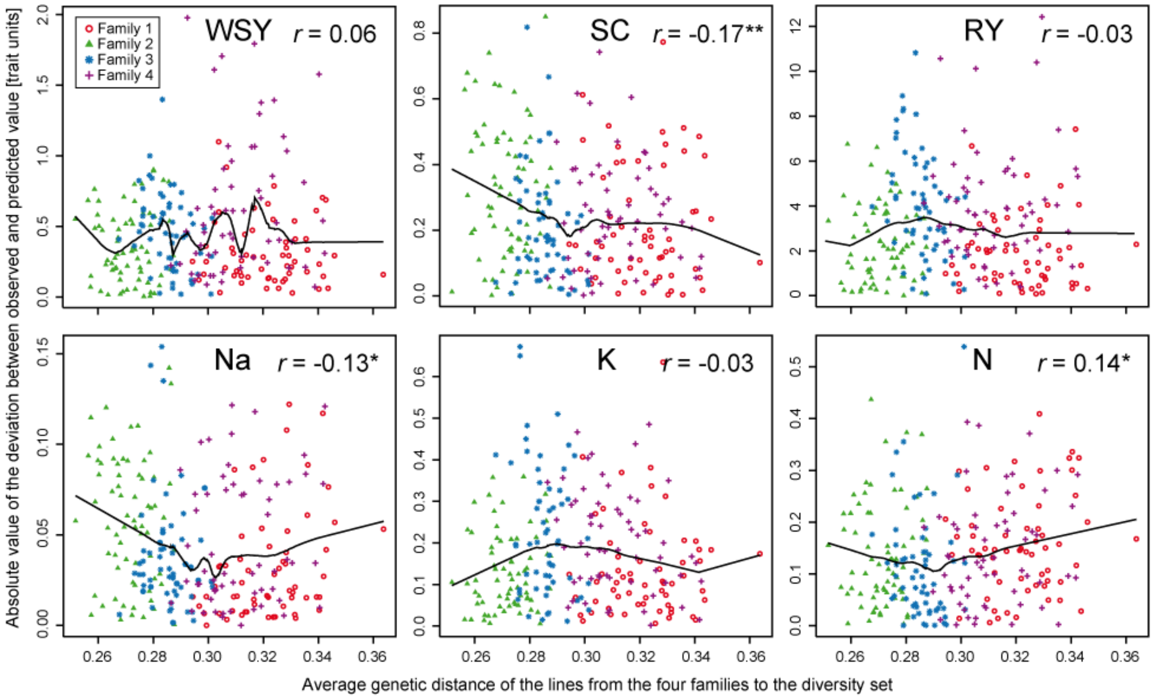

Figure S3 Association between genetic distance and prediction accuracy. Association between genetic distance (mean modified Rogers' distance between each line in the four families with all lines in the diversity set) and the absolute deviation between observed and predicted phenotypic values (for marker effects estimated in the diversity set and prediction done in the four families). Curve was fitted by robust locally fitted regression. Results are shown for white sugar yield (WSY), sugar content (SC), root yield (RY), sodium content (Na), potassium content (K), and α-amino nitrogen content (N).

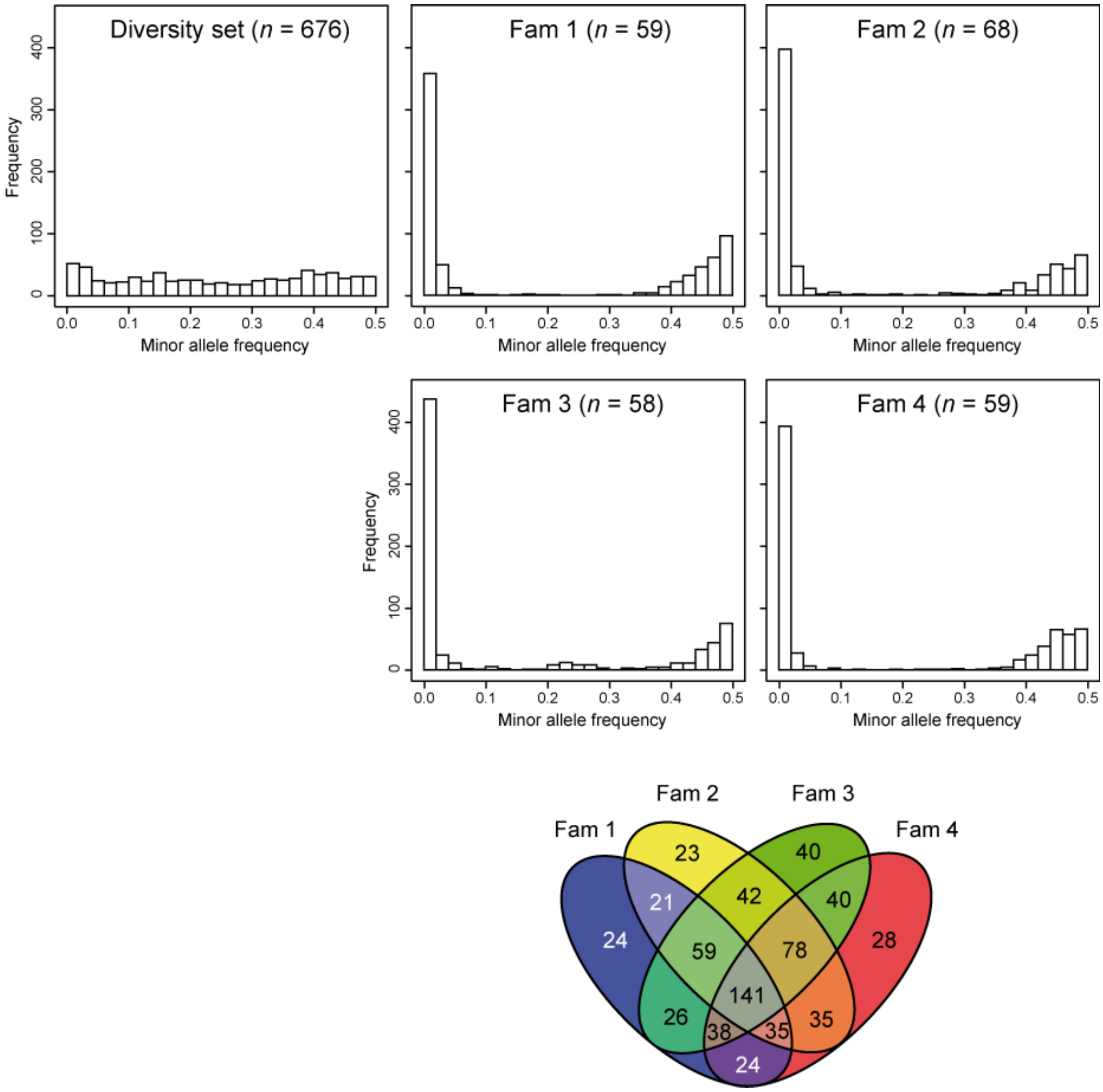

Figure S4 Minor allele frequency in the diversity set and in the four families. The Venn diagram illustrates the numbers of overlapping and family-specific monogenic markers.
